# Hypoparathyroidism, Sensorineural Deafness, and Renal Disease Syndrome Presenting With Febrile Seizures and Hypocalcemia

**DOI:** 10.1210/jcemcr/luac025

**Published:** 2023-01-09

**Authors:** Anne Gandolfi, Kevin Ratnasamy, Carla Minutti

**Affiliations:** Department of Pediatrics, Rush University Medical Center Rush Pediatric Residency Program, Chicago, IL 60612, USA; Department of Combined Internal Medicine-Pediatrics, Rush University Medical Center, Chicago, IL 60612, USA; Department of Pediatric Endocrinology, Rush University Medical Center, Chicago, IL 60612, USA

**Keywords:** Barakat syndrome, HDR syndrome, hypoparathyroidism, hypocalcemia, GATA3

## Abstract

HDR syndrome is a rare genetic disorder caused by mutations in the *GATA3* gene and characterized by hypoparathyroidism, sensorineural deafness, and renal disease. Here, we report case of a 9-month-old male with history of hydronephrosis and sensorineural deafness who presented with febrile seizures. He was found to have hypocalcemia and inappropriately normal parathyroid hormone. His neurologic and infectious workup were negative. Genetic testing revealed a nonsense mutation in the *GATA3* gene, consistent with HDR syndrome. Hypocalcemia was responsive to calcium carbonate and calcitriol treatment. This case highlights hypocalcemia caused by hypoparathyroidism as a potential etiology of seizures. When hypoparathyroidism is detected with either hearing loss or renal disease, HDR syndrome should be considered, and other features of the syndrome should be investigated.

Hypoparathyroidism, sensorineural Deafness, and Renal dysplasia (HDR) syndrome is a rare genetic disorder caused by *GATA3* haploinsufficiency that may be identified in early life with sensorineural deafness or renal anomalies, or later in life with hypocalcemia secondary to hypoparathyroidism. Besides the 3 characteristic features of hypoparathyroidism, sensorineural deafness, and renal disease, there have been other features associated with the disorder described in the literature. Here, we describe a case of HDR syndrome caused by a previously reported nonsense mutation of the *GATA3* gene associated with all 3 characteristic phenotypic features, in addition to developmental delay, dysmorphic facial features, cortical visual impairment, and febrile seizures. Because this disorder exhibits variable expressivity and incomplete penetrance, it is imperative to evaluate patients and family members for parathyroid, auditory, and renal abnormalities so that appropriate treatment may begin. This report may contribute to the body of literature in describing less-common features associated with HDR syndrome and the genetic abnormalities that most commonly cause this disease.

## Case Presentation

A 9-month-old male fraternal twin with past medical history of prematurity (born at 35 weeks 1 day gestation), developmental delay, grade 1 left hydronephrosis due to megaureter, cortical visual impairment, nystagmus, and bilateral sensorineural hearing loss with hearing aids presented in the Emergency Department (ED) with approximately 6 minutes of tonic-clonic seizure activity in the setting of fever noted at the time of presentation. He had no associated cough, congestion, vomiting, or diarrhea. He had no prior history of seizure-like activity. Dietary history was notable for lactose intolerance, and he was on lactose-free formula (Nutramigen). His family history was positive for his mother with childhood seizures and a 4-year-old brother with staring episodes. The brother was seen by a neurologist for “staring spells,” which began at 2 months of age. The brother had a normal ambulatory electroencephalogram (EEG) and magnetic resonance imaging (MRI) of the brain at 16 months of age. The frequency of the spells decreased and then stopped by 2 years of age.

## Diagnostic Assessment

In the ED, his physical exam was notable for an alert and interactive infant with horizontal nystagmus and no focal neurologic deficits. Vital signs showed blood pressure of 102/53 mmHg, heart rate of 156 beats per minute, temperature of 38.4 degrees Celsius, respiratory rate of 32, and oxygen saturation of 97%. His weight on presentation was 9.9 kg. Laboratory evaluation was significant for hypocalcemia at 7.0 mg/dL (ref. 9–11 mg/dL; 1.75 mmol/L), white blood cell count of 17.1 × 10^9^/L (17100/uL) (ref. 6.0–14 × 10^9^/L) with 74.4% neutrophils (ref. 22.0%–40.0%). A urinalysis showed white blood cells 6–10/hpf (ref. 0–5/hpf), rare bacteria, and negative leukocyte esterase. A computed tomography scan of the head and a chest radiograph were unremarkable. He was admitted to the general pediatric floor for further evaluation. Laboratory evaluations the following day showed low adjusted calcium of 7.2 mg/dL (ref. 9.2–11.2 mg/dL; 1.8 mmol/L), albumin of 3.4 g/dL (ref. 2.9–5.5 g/dL), low ionized calcium of 0.78 mmol/L (ref. 0.95–1.32 mmol/L; 3.12 mg/dL), elevated phosphorus of 8.2 mg/dL (ref. 4.5–6.5 mg/dL; 2.65 mmol/L), and inappropriately normal intact parathyroid hormone (PTH) of 18.3 pg/mL (ref. 8.0–85.0 pg/mL; 18.3 ng/L). 25-hydroxy vitamin D was 48 ng/mL (ref. > 30 ng/mL; 119.8 nmol/L). Serum magnesium was 2.4 mg/dL (ref. 1.6–2.7 mg/dL; 0.99 mmol/L). An electrocardiogram showed a prolonged QTc interval (453 ms). Parathyroid hormone antibody was sent out (Mayo Clinic, Radiobinding Assay performed by Quest Diagnostics Nichols Institute, Test ID FPTH) to evaluate for autoimmune disease of the parathyroid gland, which was negative. Neurologic workup was unremarkable, including electroencephalogram, magnetic resonance imaging of the orbits and the brain, and cerebrospinal fluid studies of glucose, protein, cell count, lactic acid, pyruvic acid, and amino acid panel. Urine culture collected in the ED grew 75 000–100 000 colony forming units (CFU) of S*taphylococcus haemolyticus*. Repeat urinalysis (which was obtained prior to initiation of any antimicrobials) and urine culture were negative. A voiding cystourethrogram (VCUG) showed bilateral grade IV vesicoureteral reflux, and retroperitoneal ultrasound showed moderate left hydroureteronephrosis.

## Treatment

Per consultation with the patient's primary urologist, ceftriaxone was administered for treatment of presumed urinary tract infection (UTI) considering urinalysis results and the patient's known urinary tract anomaly. Antibiotics were discontinued after urine culture resulted with presumed contaminant. Calcium carbonate was started at a dose of 45 mg/kg/day of elemental calcium orally divided into 3 doses for hypoparathyroidism with hypocalcemia.

## Outcome and Follow-Up

He had no seizures during hospitalization. Calcium carbonate was adjusted to 50 mg/kg/day of elemental calcium divided twice daily on hospital day 4 for less frequent medication administration. His calcium, phosphorus, and QTc normalized by hospital day 3. Due to the degree of reflux on voiding cystourethrogram, UTI prophylactic antibiotics were initiated. He was discharged home on hospital day 5 with follow-up with endocrinology, urology, and genetics and new prescriptions for calcium carbonate 50 mg/kg/day of elemental calcium and nitrofurantoin.

Outpatient genetic testing was completed due to concern for an underlying genetic syndrome with the constellation of cortical visual impairment, sensorineural hearing loss, congenital renal anomalies, and hypoparathyroidism with hypocalcemia. A buccal swab was sent for sequencing analysis (GeneDx, Autism/ID Xpanded Panel, Gaithersburg, MD), which revealed a heterozygous nonsense mutation in exon 6 of the *GATA3* gene, c.1099C>T; p.(R367*), which was presumed to be de novo since both parents tested negative for *GATA3* mutations. This loss-of-function variant was predicted to result in protein truncation of the last 78 amino acids. Due to the finding of the pathogenic genetic variant and characteristic phenotypic features, he was diagnosed with Hypoparathyroidism, sensorineural Deafness, and Renal dysplasia (HDR) syndrome, also known as Barakat syndrome. Family members did not complete calcium level tests.

Two weeks after discharge, labs were repeated to monitor serum ionized calcium levels and allow for titration of calcium carbonate dosing. Ionized calcium was 0.90 mmol/L (ref. 0.95–1.32 mmol/L; 3.6 mg/dL), magnesium was 1.9 mg/dL (ref. 1.6–2.7 mg/dL; 0.78 mmol/L), and phosphorus was 7.7 mg/dL (ref. 4.5–6.5 mg/dL; 2.49 mmol/L). Calcium carbonate was increased from 50 mg/kg/day of elemental calcium to roughly 63 mg/kg/day with twice daily dosing. One month after discharge, ionized calcium remained low at 0.85 mmol/L (3.4 mg/dL), with unchanged magnesium and phosphorus levels. Calcium carbonate was again increased to roughly 94 mg/kg/day of elemental calcium with 3 times daily dosing. Ionized calcium was repeated in a week and was 0.88 mmol/L (3.52 mg/dL). Due to persistence of hypocalcemia, calcitriol was added at a dose of 0.02 mcg/kg/day. Repeat ionized calcium 1 week after the initiation of calcitriol was normal at 1.01 mmol/L (4.04 mg/dL), with stable magnesium at 2.1 mg/dL (0.86 mmol/L) and persistently elevated phosphorus at 7.7 mg/dL (2.49 mmol/L).

## Discussion

Barakat syndrome (OMIM 146255) was first described in 2 male siblings presenting with steroid-resistant nephrotic syndrome, sensorineural deafness, and hypoparathyroidism with hypocalcemia at ages 3½ and 5 years [1]. It was later termed HDR syndrome in 1992 in a family presenting with an autosomal dominant pattern of the 3 main phenotypic features: hypoparathyroidism, sensorineural deafness, and renal disease [2].

Most cases of HDR syndrome are caused by haploinsufficiency of the *GATA3* gene on chromosome 10p14, which codes for a transcription factor that binds to a zinc finger domain [3]. *GATA3* is part of a family of highly conserved transcription factors and is important in embryonic development of the parathyroids, auditory system, kidneys, thymus, and central nervous system [3, 4]. In a review of 180 cases of HDR syndrome, 66.4% were associated with *GATA3* mutations, 7.8% had no *GATA3* mutation, and 25.9% were not tested for *GATA3* [4]. This disorder exhibits variable expressivity, incomplete penetrance, and autosomal dominant inheritance.

Although there are no defined diagnostic criteria for HDR syndrome, it has been suggested that the diagnosis can be made in patients with all 3 phenotypic features or 2 phenotypic features and a positive family history [4]. In patients with isolated and unexplained sensorineural deafness or renal disease with a positive family history, *GATA3* testing should be completed to make a diagnosis [4].

Only 64.4% of patients present with all 3 phenotypic features, with hearing loss as the most common feature (96.7%), followed by hypoparathyroidism (93.3%) and renal disease (72.2%) [4]. With the advent of newborn hearing screens and routine fetal ultrasounds, many patients are identified with hearing loss or structural kidney anomalies early in life. Less commonly, patients will be identified later in life with signs and symptoms of hypocalcemia secondary to hypoparathyroidism, such as seizures or tetany (ie, Chvostek's and Trousseau's signs). Seizures associated with hypocalcemia are usually generalized tonic-clonic and are more likely to be caused by rapid changes in serum calcium rather than a particular calcium level [5]. Parathyroid hormone levels may range from undetectable to inappropriately normal. Postmortem evaluation of patients has revealed parathyroid glands with hypoplasia, fibrosis, and fatty infiltration [1]. Highly variable renal abnormalities have been identified, including bilateral or unilateral structural anomalies (dysplasia, cysts, vesicoureteral reflux) and functional abnormalities (nephrosis, renal tubular acidosis, nephrocalcinosis) [3, 6]. Although there are no current guidelines on renal imaging for HDR syndrome, it would likely be helpful to perform renal ultrasonography at the time of diagnosis to evaluate for structural anomalies. Other less-common features that have been described are hypogonadotropic hypogonadism, polycystic ovaries, female genital tract anomalies, dysmorphic features, congenital heart disease, cognitive disability, choanal atresia, biliary atresia, hemimegalencephaly, hypomagnesemia, and retinitis pigmentosa [4, 7]

HDR syndrome is managed symptomatically based on the presenting features. Hypoparathyroidism should be treated with elemental calcium 30 to 75 mg/kg/day and calcitriol 0.02 to 0.06 mcg/kg/day, with adjustment based on close monitoring of serum calcium, urine calcium, and serum phosphorus levels. There are no current guidelines regarding the frequency of biochemical monitoring for pediatric hypoparathyroidism with hypocalcemia; however, we suggest monitoring serum calcium levels weekly until stable, and then spacing to every 2 to 3 months. Special attention should be paid to laboratory monitoring in patients with extensive renal disease to prevent overtreatment and the development of nephrocalcinosis. Oral therapy with outpatient monitoring is appropriate for most patients who are asymptomatic, while hypocalcemia resulting in mental status changes or seizures should be managed with IV calcium and inpatient monitoring [5].

From the literature, it remains unclear when the diagnosis is typically made, likely due to lack of standard diagnostic criteria and low disease incidence. Deafness is the earliest identified symptom at an average age of 7.5 years, followed by renal disease (14 years) and hypoparathyroidism (15.3 years) [7]. Although the presence and severity of each phenotypic feature may vary at time of diagnosis, nearly all patients will manifest all 3 characteristics by the age of 50 [7]. The prognosis is typically dependent on the extent of renal disease.

Here, we describe a case of HDR syndrome caused by nonsense mutation of the *GATA3* gene associated with all 3 characteristic phenotypic features, in addition to developmental delay, dysmorphic facial features, cortical visual impairment, and febrile seizures. This report may contribute to the body of literature in describing less-common features associated with HDR syndrome and the genetic abnormalities that most commonly cause this disease.

## Learning Points

- In patients with unexplained hypoparathyroidism associated with a history of sensorineural deafness or renal disease, HDR syndrome should be considered with appropriate investigation for all 3 characteristic features for the patient and family members.
- HDR syndrome exhibits variable expressivity and incomplete penetrance, but nearly all patients will exhibit the 3 characteristic features by the age of 50. Prognosis is dependent on the extent of renal disease.
- *GATA3* genetic testing should be performed in patients with unexplained sensorineural deafness or renal disease with a positive family history of HDR syndrome. Evaluation of hypoparathyroidism should also be performed to ensure subclinical hypocalcemia does not go undetected.

## Data Availability

Data sharing is not applicable to this article as no datasets were generated or analyzed during the current study.

## Abbreviations

ED: Emergency Department
HDR syndrome: hypoparathyroidism, sensorineural deafness, and renal disease
UTI: urinary tract infection
